# Synergy or Dominance? The Ergogenic Effects of Caffeine and Carbohydrate on High-Intensity Interval Exercise Performance: A Three-Level Meta-Analysis

**DOI:** 10.3390/nu18121868

**Published:** 2026-06-10

**Authors:** Hao Li, Yixiang Peng, Baiyu Liu, Li Ding, Kai Xu, Tze-Huan Lei, Bomin Gong, Yinhang Cao

**Affiliations:** 1 School of Athletic Performance, Shanghai University of Sport, Shanghai 200438, China; lihao053119@163.com (H.L.); lidingsus@163.com (L.D.); 2221152066@sus.edu.cn (K.X.); 2 Faculty of Health Sciences and Sports, Macao Polytechnic University, Macao 999078, China; p2525351@mpu.edu.mo; 3 School of Physical Education, Shanghai Normal University, Shanghai 201418, China; 19276322688@163.com; 4 College of Physical Education, Hubei Normal University, Huangshi 435002, China; tzehuanlei@gmail.com; 5 Department of Physical Education, Shanghai University of Traditional Chinese Medicine, Shanghai 201203, China

**Keywords:** high-intensity interval exercise, caffeine, carbohydrate, ergogenic aids

## Abstract

**Background/Objectives**: This meta-analysis aimed to quantify the combined ergogenic effects of caffeine (CAF) and carbohydrate (CHO) on high-intensity interval exercise (HIIE) performance and to identify potential moderating factors. **Methods**: Four databases were systematically searched to identify randomized crossover trials assessing CAF combined with CHO (CAF + CHO) on HIIE performance (i.e., exercise time, distance, or total work). A three-level random-effects meta-analysis was performed to calculate pooled Hedge’s *g* (g) values. Moderator effects were explored through subgroup analyses, including control group (placebo-, CHO-, and CAF-controlled), CHO administration (mouth rinse and ingestion), and training status (recreationally active and trained). **Results**: Eleven studies met the inclusion criteria (*n* = 105; 8 female). CAF + CHO significantly enhanced HIIE performance (g = 0.44, 95% CI: 0.23–0.66). Subgroup analyses indicated that CAF + CHO mouth rinsing (g = 0.91, CI: 0.49–1.33) yielded superior effects compared to CAF + CHO ingestion (g = 0.33, CI: 0.14–0.52) (*p* for subgroup < 0.05). Performance improvements with CAF + CHO were observed for CHO- and placebo-controlled trials, but not in CAF-controlled trials, without significant subgroup effects (*p* for subgroup > 0.05). Importantly, evidence of publication bias was identified, and the overall certainty of evidence was graded as low according to the GRADE framework. **Conclusions**: CAF + CHO appears to be effective for enhancing HIIE performance, with greater benefits observed when CHO is administered via mouth rinsing rather than ingestion. Preliminary evidence suggests that CAF may play a key role in CAF + CHO strategies. However, given the limited number of female participants, the generalizability of these findings to both sexes is limited. Additional high-quality trials are needed to establish more definitive recommendations.

## 1. Introduction

Intermittent sports (e.g., team, racket, and combat sports) are characterized by repeated bouts of high-intensity activity (e.g., repeated approach spike jump, consecutive high-intensity forehand drives, or tackling and jumping) interspersed with brief recovery periods over prolonged durations [1,2,3]. The ability to sustain high-intensity interval exercise (HIIE) performance is critical for success in these sports [4]. Accordingly, coaches and athletes are increasingly exploring nutritional supplementation strategies to enhance HIIE performance of intermittent sport athletes, thereby facilitating improvements in competitive performance.

Caffeine (CAF) and carbohydrate (CHO) are widely used ergogenic aids [5,6]. Previous studies have demonstrated the ergogenic effects of CAF on endurance [7,8], resistance [9,10] and HIIE performance [11,12]. CAF acts mainly through antagonism of adenosine receptors, thereby affecting central and peripheral physiological systems [13]. Additionally, CHO exerts ergogenic effects when ingested by serving as a primary energy source for high-intensity exercise and maintaining glycogen stores and blood glucose levels [14], or via CHO mouth rinsing (CMR), which stimulates oral receptors and activates brain regions related to reward and motor control [15]. Therefore, CAF combined with CHO (CAF + CHO) may exert complementary effects by targeting both metabolic and neural pathways, thereby producing additive benefits and maximizing HIIE performance.

Recent research has investigated the impact of combined CAF + CHO supplementation on HIIE performance. Nevertheless, the findings remain inconsistent. For example, performance improvements have been observed with CAF + CHO mouth rinsing in trained individuals, whereas no significant benefit has been reported with CAF + CHO ingestion in recreationally active individuals. These discrepancies are potentially due to supplementation protocols (e.g., administration mode) and participant characteristics (e.g., training status) [16,17].

Meta-analysis increases the ability to identify meaningful effects by pooling data across studies, providing more precise estimates and a stronger basis for synthesizing evidence than single trials alone [18]. Meanwhile, the absence of subgroup and meta-regression analyses limits assessment of participant or intervention characteristics as effect modifiers. For example, since CMR acts via central neural drive rather than substrate oxidation [16,18], its combination with CAF may exhibit distinct synergistic effects compared to CHO ingestion. Furthermore, previous studies have demonstrated that training status modulates the efficacy of CAF, with trained individuals deriving greater CAF ergogenic benefits [17]. This highlights the need to quantify the overall ergogenic effect of CAF + CHO on HIIE performance and examine moderators explaining variability in the results.

Thus, we employed a three-level meta-analytic model to synthesize randomized controlled trials examining CAF + CHO supplementation (via ingestion or CMR) during HIIE. This advanced modeling approach was specifically chosen to appropriately handle the correlation among multiple effect sizes that are often reported in the same participant cohort. We also performed moderator analyses to assess how supplementation protocols and participant characteristics influence ergogenic efficacy. Specifically, we hypothesized that CAF + CHO would enhance HIIE performance, with effects varying by protocol and participant characteristics.

## 2. Materials and Methods

This review was conducted in accordance with the PRISMA 2020 reporting standards for systematic reviews and meta-analyses [19]. This review was prospectively registered with PROSPERO (registration number: CRD420261280614).

### 2.1. Literature Search

Four major databases (Web of Science, the Cochrane Library, PubMed, and Scopus) were systematically searched for relevant literature from inception to 25 April 2026. The search strategy combined controlled vocabulary with free-text keywords using Boolean operators (“AND,” “OR,” “NOT”) and truncation symbols to identify studies related to “caffeine,” “carbohydrate,” and “high-intensity interval exercise” (see Supplementary S1 for search details). In addition, PROSPERO was searched to verify that no similar systematic review protocol had already been registered, and reference lists of relevant review articles were searched manually to retrieve additional studies.

### 2.2. Eligibility Criteria

Study eligibility was determined using prespecified inclusion and exclusion criteria structured following the PICOS framework: (1) eligible participants were healthy adults aged 18 years or older; (2) studies including an intervention protocol using CAF + CHO ingestion or CAF + CMR administered before and/or during HIIE—eligible HIIE protocols consisted of per repetition lasting ≤ 8 min, separated by recovery periods ≤ 300 s, performed at ≥90% of maximal oxygen uptake or involving “all-out” maximal efforts [20]; (3) inclusion of at least one comparison condition using CHO or placebo (PLA) or CAF control; (4) reporting at least one HIIE performance outcome (i.e., exercise time, distance, peak power [PP], mean power [MP], or total work); and (5) adopted a randomized, double-blind crossover design.

The following exclusion criteria were applied: (1) articles published in languages other than English; (2) studies evaluating prolonged CAF + CHO co-supplementation in the context of HIIE; (3) review articles, case-study reports, publications that were not peer-reviewed, and conference papers; (4) animal studies; and (5) research primarily concerned with clinical treatment or disease conditions.

### 2.3. Study Screening and Selection

Duplicate records were removed manually by one reviewer (H.L.) using EndNote X9 (Clarivate Analytics, Philadelphia, PA, USA). Two investigators (H.L. and Y.P.) independently reviewed titles and abstracts for eligibility. Differences were reconciled via discussion, with a third reviewer (L.D.) involved when agreement could not be reached. Subsequently, the aforementioned investigators separately evaluated the complete manuscripts of potentially relevant studies. Eligible studies were selected according to the predefined criteria.

### 2.4. Quality Assessment and Risk of Bias

Methodological rigor was evaluated with the Cochrane Risk of Bias framework, as implemented within Review Manager (RevMan) 5.4 (The Nordic Cochrane Centre, 2014) [21]. The evaluation encompassed core domains such as sequence generation, allocation concealment, blinding, incomplete outcome data, selective reporting, and other bias (within which crossover-specific biases, including the adequacy of washout periods and carry-over effects, were rigorously evaluated). Each domain was assessed as “low” (“+”), “high” (“−”), or “unclear” (“?”) risk of bias. The assessment was carried out independently by two reviewers (H.L. and Y.P.). Disagreements were resolved through discussion. When necessary, a third reviewer (B.L.) made the final judgment. We further assessed methodological quality with the Physiotherapy Evidence Database (PEDro) scale [22]. High quality was defined by a score of 6 or greater, whereas moderate and low-quality ratings were assigned to studies achieving 4–5 and 3 or fewer points, respectively.

### 2.5. Statistical Analyses

#### 2.5.1. Data Collection and Study Categorization

Data were extracted independently by two investigators (H.L. and Y.P.) using a Microsoft Excel (Version 16.93; Microsoft Corp., Redmond, WA, USA). They collected information on study design, sample characteristics (e.g., sample size, sex, training background), exercise regimens, and HIIE performance outcomes (including duration, distance, total work, and both mean and peak power output). Any discrepancies were resolved through discussion. When numerical data were missing or only presented in figures, values were obtained using WebPlotDigitizer (Version 4.7) [23]. In cases with multiple relevant outcomes, the results were merged into a single averaged mean, and standard errors were converted to standard deviations according to Cochrane guidance [24].

Differences in HIIE performance between the CAF + CHO and control conditions were examined to evaluate supplementation effects. The computation of the mean difference (MD) and the SD of the change (${SD}_{pooled}$) was executed based on the protocols of the Cochrane Handbook (Version 6.5, 2024) utilizing equation [25]. First, the difference between the means was computed:

(1) $$MD=M_{CO}-M_{PLA}$$

In this equation, $M_{CO}$ denotes the mean value observed in the CAF + CHO condition, whereas $M_{PLA}$ denotes the mean value observed in the PLA condition.

The pooled standard deviation of the mean differences (${SD}_{pooled}$) for crossover studies was then computed using the method described below [26]:

(2) $${SD}_{pooled}=\sqrt{\frac{{SD}_{CO}^{2}+{SD}_{PLA}^{2}}{2}}$$

where ${SD}_{CO}$ is the standard deviation from CAF + CHO group and ${SD}_{PLA}$ is the standard deviation from PLA group.

Given that most included studies had small sample sizes, we used Hedge’s *g* (g) as the effect size metric in all analyses. For crossover designs, *g* was calculated as follows [27]:

(3) $$H\mathrm{edge}’s\;g=\frac{M_{CO}-M_{PLA}}{{SD}_{pooled}}\times (1-\frac{3}{4(N-1)-1})$$

where N denotes the overall number of participants.

The standard error (SE) of the effect estimate *g* for crossover trials was determined as follows [27]:

(4) $$SE=\sqrt{\frac{1}{N}+\frac{g^{2}}{2N}}\times \sqrt{2(1-r)}$$

where r denotes the assumed within-participant correlation between the CAF + CHO and control conditions. Because this correlation was seldom documented across the incorporated crossover trials and was not provided in a previous meta-analysis in this area, we followed Cochrane Handbook guidance [24] and set r = 0.50 for the primary analysis. To evaluate the sensitivity of the results to this assumption, we repeated the analysis using alternative values (r = 0.20 and r = 0.80) when pooling overall outcomes.

#### 2.5.2. Meta-Analytic Methods and Heterogeneity Assessment

A three-level meta-analysis was conducted in accordance with the recommendations of Assink and Wibbelink [28] to handle the statistical dependence between effect sizes reported within the same study (e.g., MP, PP, and total work) and to avoid double counting or ignoring within-study correlations [29]. Using this approach, variance is separated into three components—sampling variance (Level 1), within-study variance (Level 2), and between-study variance (Level 3), which allows for correlated effect sizes and a nested data structure [30]. We fitted the three-level model using REML, and then refit it using ML to examine the stability of the findings. Hedges’ g was interpreted using conventional SMD thresholds was categorized as *trivial*, *small*, *moderate*, or *large* for values of <0.20, 0.20–0.49, 0.50–0.79, and ≥0.80, respectively [24].

We used the t-distribution [31] to test individual coefficients and to derive the corresponding 95% confidence intervals (CIs) across all models. Prediction interval (PI) was also calculated to provide additional insight into the dispersion of true effects under the random-effects model [32]. Across-study variability was quantified using *I*^2^ and the prediction interval (PI) and categorized as *low*, *moderate*, *substantial*, or *considerable* within the ranges of 0–25%, 25–50%, 50–75%, and 75–100%, respectively [24]. Statistical significance was set at *p* < 0.05.

To evaluate heterogeneity sources and potential moderators, subgroup analyses were performed based on categorical variables [33]. Subgroup analyses were conducted if three or more eligible studies were identified [24]. We performed subgroup analyses based on the following variables: (a) administration methods; (b) control group; and (c) training status.

Administration methods were classified as CHO ingestion or CMR, controls as PLA-, CHO-, or CAF-control, and training status as recreationally active or trained based on established criteria [34]. Analyses were conducted in R (V.4.2.0, R Core Team, Vienna, Austria) [35] using metafor (version 4.8-0), with visualization using ggplot2 (version 4.0.1) and orchaRd (version 2.1.3) [36]. Power analyses were performed for each subgroup as well as the overall pooled effect to limit the chance of false negatives.

#### 2.5.3. Reporting Bias Assessment and Robustness Checks

Potential reporting bias was assessed by visually inspecting funnel plots [37] and by applying Egger’s regression test [38], and the trim-and-fill method. These analyses were conducted only when at least 11 studies contributed to an outcome (k > 10) [39]. When *p* exceeded 0.05, publication bias was considered not evident.

We conducted leave-one-out sensitivity analyses to verify the reliability of pooled estimates in the three-level meta-analysis, while the impact of varying correlation coefficients on SEs was examined. Influential or outlying effect sizes at Level 2 (within-study) and Level 3 (between-study) were diagnosed using Cook’s distances values [40] and studentized residuals [41]. Observations were flagged when hat values or Cook’s distances values were greater than three times the corresponding mean values, or when the absolute studentized residual was >2. The model was then re-estimated after excluding flagged effect sizes to determine whether the pooled estimates were materially affected.

### 2.6. Certainty of the Evidence

The certainty of the evidence was judged using the GRADE approach (Grading of Recommendations Assessment, Development and Evaluation) when interpreting the results. We assigned one of four certainty categories (*high*, *moderate*, *low*, or *very low*), reflecting progressively reduced confidence that the estimated effect is close to the true effect. Specifically, a *high* rating indicates that further studies are unlikely to materially alter the estimate; *moderate* suggests that additional research could meaningfully affect confidence and may change the estimate; *low* indicates that new evidence is expected to substantially influence confidence and is likely to change the estimate; and *very low* denotes very limited confidence in the estimate, with considerable uncertainty surrounding the effect [42]. GRADE ratings were completed by one reviewer (H.L.) and cross-checked independently by a second reviewer (Y.P.). The reviewers discussed discrepancies and reached agreement by consensus.

## 3. Results

### 3.1. Selection of Studies and Characteristics

**Figure 1:**
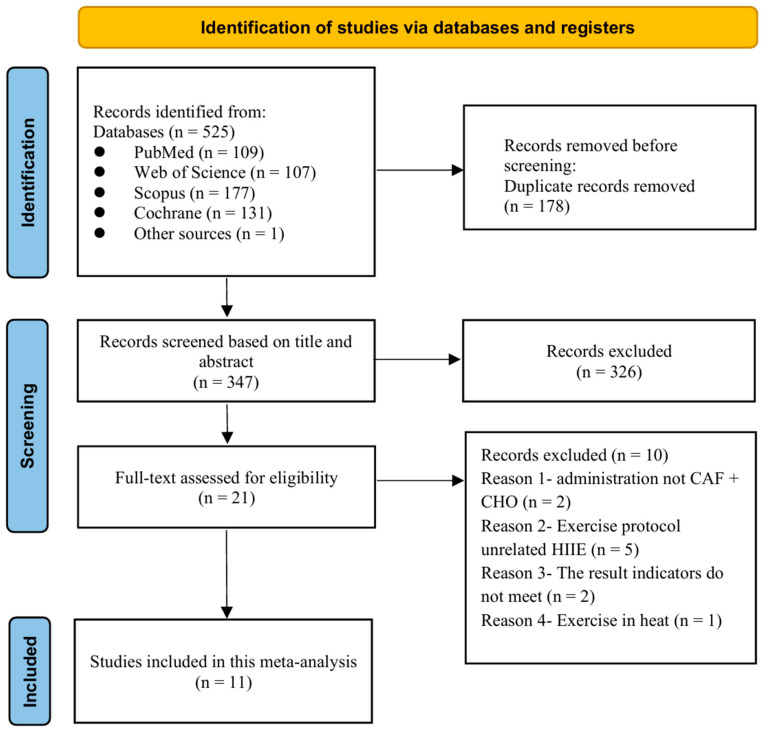
Flow diagram of study selection.

The database search yielded 525 records initially, comprising 524 from electronic databases and 1 from additional sources. Following screening and eligibility evaluation, the meta-analysis ultimately included eleven studies [43,44,45,46,47,48,49,50,51,52,53], yielding 30 data points in total. See Figure 1 for the study selection process.

Sample sizes across studies varied from 6 to 13, yielding a combined total of 105 participants. Mean participant age ranged from 21 to 32 years. Overall, 44% were classified as recreationally active athletes (k = 14) and 56% as trained (k = 16). Regarding supplementation strategies, 9 studies (k = 26) examined CAF + CHO ingestion, while two studies (k = 4) investigated CAF + CMR. Further study details are presented in Table 1.

### 3.2. Evaluation of Methodological Rigor and Potential Bias

**Figure 2:**
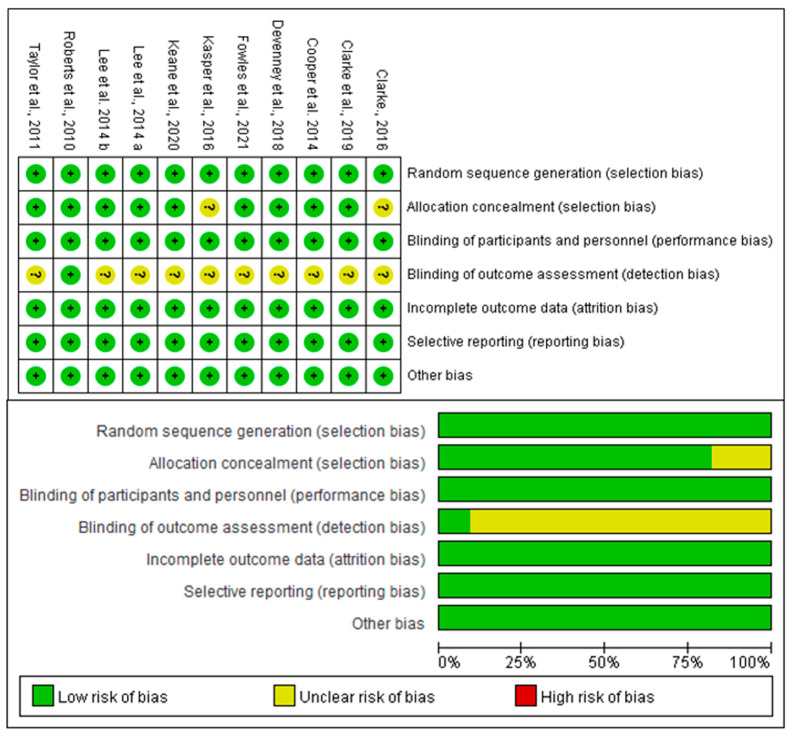
Quality appraisal of eligible publications.

Figure 2 summarizes the bias risk evaluations for all included studies. Allocation concealment was insufficiently described in two studies [45,53], leading to a “some concerns” judgment for this domain. Only one study [52] assessed the effectiveness of blinding, while the remaining articles were classified as “some concerns” for blinding of outcome assessment. Furthermore, all included crossover trials were assessed as low risk of carry-over effects within the “other bias” domain, as adequate washout periods (e.g., 5–7 days) were implemented between experimental conditions.

The 11 studies had PEDro scores in the range of 8–11. (Supplementary S2), with a mean of 8.9, reflecting generally good methodological quality. Nine studies were rated as good, and two as excellent. Notably, only one study reported the effectiveness of blinding of experimental conditions (item 12).

Statistical evidence from Egger’s test revealed possible asymmetry indicative of small-study effects for overall HIIE performance (*p* < 0.05). Nevertheless, the trim-and-fill procedure yielded a still-significant pooled estimate (Supplementary S4), indicating that the primary conclusions were unlikely to be materially influenced by publication bias. For moderator analyses, Egger’s regression was applied to subgroups with at least 10 studies (k ≥ 10). Funnel-plot asymmetry was observed in the eligible subgroups (*p* < 0.05), indicating a possible bias risk.

### 3.3. Primary Results

**Figure 3:**
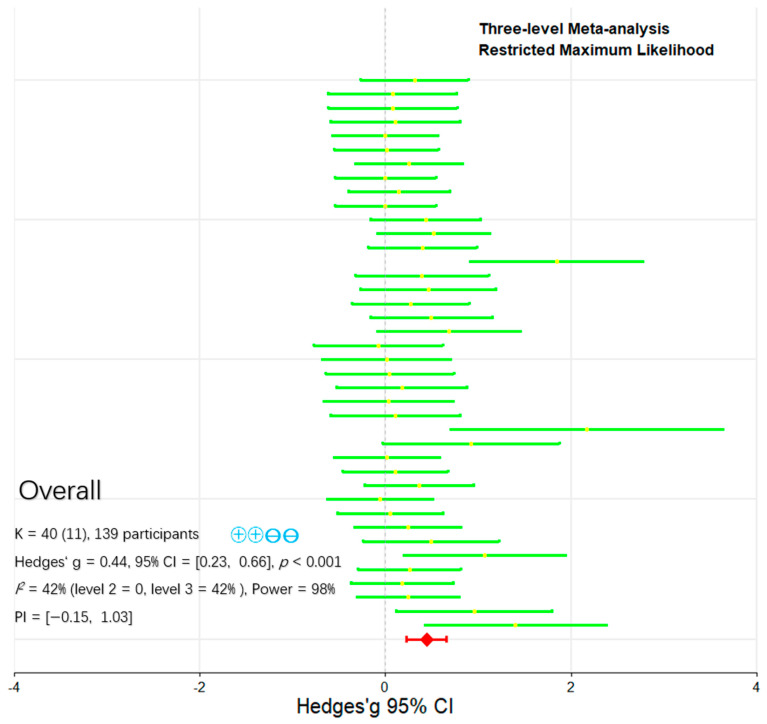
Primary pooled effects of CAF + CHO on overall performance during HIIE. K, count of effect estimates incorporated into the aggregate result; Hedges’ *g*, standardized effect size metric; 95% CI, 95% confidence interval; *p*-value, significance level for the pooled effect; PI, prediction interval; *I*^2^, heterogeneity statistic; Power, statistical power of the pooled effect; blue circles, GRADE rating used to appraise certainty of evidence and strength of recommendations.

In the analysis of overall HIIE performance, the three-level model suggested small-to-moderate performance improvements with CAF + CHO (k = 40, *g* = 0.44, 95% CI: 0.23 to 0.66, *I*^2^ = 42% [*moderate*], PI: −0.15 to 1.03, *p* < 0.001, *Low* GRADE) (Figure 3). Variance decomposition across the three-level model indicated no within-study component (Level 2 = 0%). Total heterogeneity originated primarily from differences occurring across research reports (Level 3 = 41%), while sampling error accounted for 58% of the entire variance (Level 1). According to Hunter and Schmidt [54], substantial heterogeneity is present when sampling error accounts for <75% of total variance. Consequently, moderator analyses were performed to investigate potential contributors to heterogeneity.

### 3.4. Moderator Analysis

Moderator analyses indicated that CAF + CHO significantly enhanced performance via both CMR (k = 4, *g* = 0.91, 95% CI: 0.49 to 1.33, *I*^2^ = 0% [low], *p* < 0.001, *Low* GRADE) and CHO ingestion (k = 36, *g* = 0.33, 95% CI: 0.14 to 0.52, *I*^2^ = 26% [*moderate*], *p* < 0.001, *Low* GRADE). Additionally, the subgroup analysis revealed a significant difference in effect sizes across CHO administration methods (*p* for subgroup = 0.031). Furthermore, the ergogenic effect of CAF + CHO ingestion was significantly lower than that of CAF + CMR.

Regarding control groups, CAF + CHO significantly improved overall HIIE performance when compared with CHO group (k = 14, *g* = 0.32, 95% CI: 0.10 to 0.53, *I*^2^ = 22% [*low*], *p* = 0.004, *Low* GRADE) and compared with PLA group (k = 16, *g* = 0.66, 95% CI: 0.29 to 1.03, *I*^2^ = 61% [*substantial*], *p* < 0.001, *Low* GRADE), but not when compared with the CAF group (k = 10, *g* = 0.10, 95% CI: −0.09 to 0.28, *I*^2^ = 0% [*low*], *p* = 0.313, *Low* GRADE). Additionally, no significant subgroup differences were observed across control groups (*p* for subgroup = 0.154).

**Figure 4:**
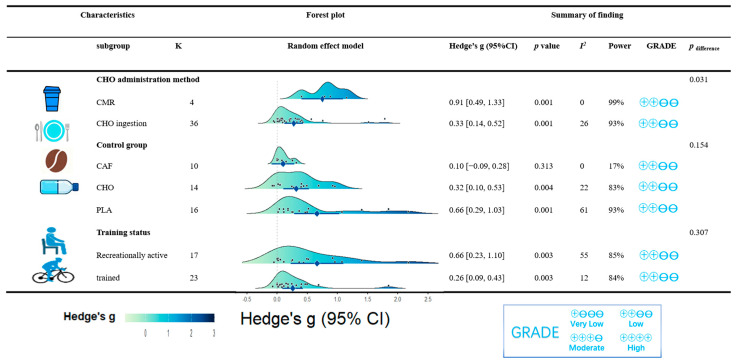
Moderator analyses evaluating the influence of CAF + CHO supplementation on total HIIE capacity by CHO administration mode, interval type, and control condition. 95% CI, 95% confidence interval; Hedges’ *g*, standardized effect size metric; *I*^2^, heterogeneity statistic; K, number of effect sizes included in the pooled estimate; *p* value, *p* value for the pooled effect estimate; *p* difference, *p* value for between-subgroup comparisons; SMD, standardized mean difference; CHO, carbohydrate; CMR, CHO mouth rinsing; CAF, caffeine; PLA, placebo.

Moderator analyses indicated that CAF + CHO improved performance in both recreationally active athletes (k = 17, *g* = 0.66, 95% CI: 0.23 to 1.10, *I*^2^ = 55% [*substantial*], *p* = 0.003, *Low* GRADE) and trained athletes (k = 23, *g* = 0.26, 95% CI: 0.09 to 0.43, *I*^2^ = 12% [*low*], *p* = 0.003, *Low* GRADE). No statistically significant difference was observed between groups when comparing subgroups defined by training status (*p* for subgroup = 0.307) (Figure 4).

### 3.5. Sensitivity Analyses

#### 3.5.1. Sensitivity Analyses of the Primary Effect

To evaluate the reliability of the primary effect estimate, we performed sensitivity analyses for crossover trials using two alternative assumptions for the within-subject correlation (r): a lower (r = 0.20) and a higher (r = 0.80) value. The overall effect size remained consistent in direction. Varying the assumed within-study correlation did not change the heterogeneity estimates, suggesting that heterogeneity was largely insensitive to the choice of correlation coefficient. Moreover, estimates derived from the ML approach were comparable to those obtained with REML, with no meaningful differences observed.

**Figure 5:**
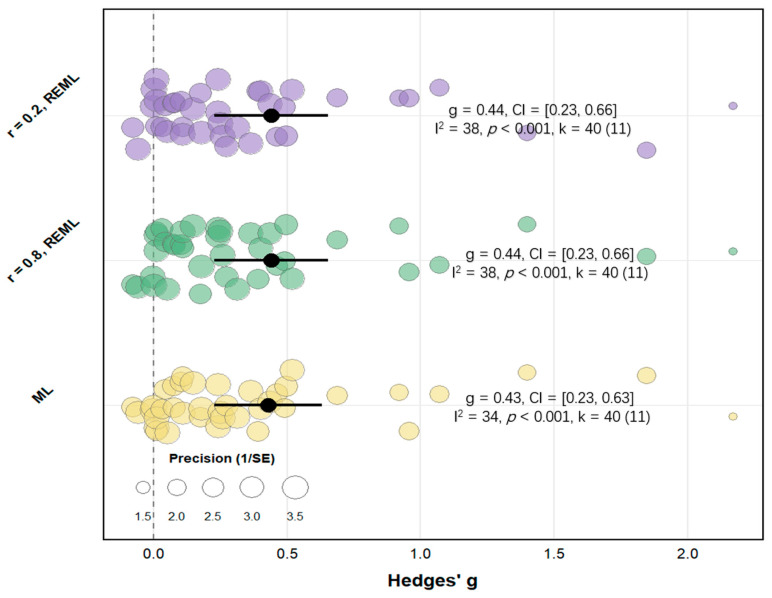
Sensitivity analyses regarding the primary results. K denotes the number of effect sizes included in the pooled estimate; Hedges’ *g* is the standardized effect size metric; 95% CI indicates the 95% confidence interval; *p*-values are reported for pooled estimates; r represents the assumed within-participant correlation between the CAF + CHO and control conditions; ML indicates the maximum-likelihood model; and *I*^2^ quantifies heterogeneity.

We screened for outliers within the three-level meta-analysis by examining Cook’s distance values and studentized residuals. Based on studentized residuals, Taylor et al. [48], Devenney et al. [43] and Kasper et al. [45] were identified as outlying studies. Additionally, Clark et al. [53] was identified as an influential observation according to both Cook’s distance values and studentized residuals. Nevertheless, excluding these studies had little effect on the updated pooled estimates (k = 36, *g* = 0.27, 95% CI 0.13 to 0.41, *I*^2^ = 12% [*low*], *p* < 0.001, PI 0.02 to 0.51, power = 96%) (Figure 5). Specifically, heterogeneity decreased after excluding the outliers, which indicated that these studies were an important source of heterogeneity.

Finally, we applied leave-one-out sensitivity analyses to the three-level meta-analysis at Level 2 (within-study) and Level 3 (between-study). All *p*-values remained significant after removing any study (*p* < 0.05). All findings indicated that the pooled results for overall HIIE performance were robust.

#### 3.5.2. Sensitivity Analyses of the Moderator Effect

Outlier diagnostics within the three-level meta-analytic framework revealed notable changes in several moderator analyses following the exclusion of influential cases. However, removing outlying values flagged by Cook’s distance values and studentized residuals did not materially change the pooled estimates in the moderator analyses (Supplementary S7).

We further assessed robustness by applying a leave-one-out approach to all moderator analyses. The results indicated that *p* values remained statistically remained significant after omitting any study (*p* < 0.05), indicating that the pooled results for all moderator groups were robust (Supplementary S6).

## 4. Discussion

We performed a meta-analysis to synthesize and assess current evidence on whether CAF + CHO improves HIIE performance and to examine potential moderators related to supplementation protocols and training status.

Our analyses indicate that CAF + CHO produces a significant improvement in HIIE performance (Figure 3). Nevertheless, the size and reproducibility of this benefit may vary across studies, likely depending on moderators such as training status and methods of CHO administration. Additionally, differences in control groups (e.g., CHO and PLA control) have also been found to potentially affect the observed results.

### 4.1. CHO Administration Method

The CHO administration methods significantly influenced the CAF + CHO, as indicated in the subgroup analysis (*p* for subgroup = 0.031) (Figure 4). Both CMR and CHO ingestion protocols yielded significant benefits of CAF + CHO. This preliminary finding suggests that both CHO ingestion and CMR forms are effective for improving HIIE performance when combined with CAF. However, CAF + CMR (*g* = 0.91, 95% CI: 0.49 to 1.33) demonstrated a significantly superior effect compared to CAF + CHO ingestion (*g* = 0.33, 95% CI: 0.14 to 0.52) (Figure 4). Mechanistically, CAF is able to traverse the blood–brain barrier to antagonize adenosine receptors and activate the central nervous system, while oral CMR simultaneously stimulates reward-related brain regions (e.g., anterior cingulate cortex, ventral striatum) via non-sweet taste receptors [6]. Furthermore, unlike traditional CHO ingestion which may induce performance-impairing gastrointestinal discomfort [55], CMR delivers a central ergogenic stimulus without systemic fluid or caloric loads. Together, these mechanisms may explain the superior efficacy of CMR. Notably, Deng et al. [18] demonstrated that CMR remains ergogenic in the fed state and improves both exercise and cognitive performance, reinforcing the value of CMR as a convenient alternative or adjunct to conventional CHO ingestion when combined with CAF.

Despite potential sample size limitations, sensitivity analyses supported the relative stability of our findings (Supplementary Material S6). Notably, excluding statistical outliers increased the CMR effect size, and leave-one-out procedures yielded consistent results, supporting the potential efficacy of CAF + CMR as an ergogenic strategy. In conclusion, while both administration forms appear to be beneficial, preliminary evidence suggests that CAF + CMR may offer a greater advantage for HIIE.

### 4.2. Control Group

Our moderator analyses revealed that CAF + CHO significantly improved HIIE performance when compared with PLA (*g* = 0.66, 95% CI: 0.29 to 1.03) (Figure 4), confirming the robust ergogenic efficacy of CAF + CHO against an inert control. Furthermore, CAF + CHO maintained a significant performance advantage when compared to CHO alone (*g* = 0.32, 95% CI: 0.10 to 0.53) (Figure 4). This finding aligns with previous meta-analytical evidence (*g* = −0.48, 95% CI: −0.85 to −0.05) [56], indicating that this advantage is likely driven by the effect of CAF. Crucially, comparing CAF + CHO with CAF alone revealed no significant additional benefit (*g* = 0.10, 95% CI: −0.09 to 0.28), suggesting that CAF acts as the primary driver in this context. Importantly, all studies in this subgroup utilized CHO ingestion over CMR, consistently reporting comparable performance benefits between CAF alone and CAF + CHO [44,46,47,53]. Thus, preliminary evidence indicates that the central neural effects of CAF may act as the potential key pathway in CAF + CHO ingestion strategies. This effect may be attributed to the pivotal role of the central nervous system in athletic performance [57]. It is widely recognized that CAF acts as an adenosine receptor antagonist [58], and promotes the secretion of *β*-endorphins and dopamine [59], which collectively mitigate central fatigue and sustain neural drive. Importantly, available evidence suggests that caffeine ingestion alone does not appear to delay gastric emptying or impair rehydration during exercise [60]. By contrast, hypertonic glucose–electrolyte solutions slow gastric emptying and shift circulatory fluid into the gut, impairing rehydration [61]. These physiological effects may partially offset the metabolic benefits of CHO ingestion during HIIE, which could help explain why CHO may play a supportive rather than primary role. Interestingly, lower-dose, fractionated carbohydrate gel ingestion within CAF + CHO strategies may enhance performance while minimizing gastrointestinal discomfort, thereby mitigating issues linked to hypertonic solutions. This suggests that CHO’s ergogenic effect may be influenced by dosage and formulation [62]. Nevertheless, considering the low certainty of the evidence and the heterogeneity identified, these results should be interpreted with caution.

### 4.3. Training Status

Our moderator analyses indicated that CAF + CHO appeared to improve HIIE performance in both recreationally active athletes (*g* = 0.66, 95% CI: 0.23 to 1.10) and trained athletes (*g* = 0.26, 95% CI: 0.09 to 0.43) (Figure 4). Although the subgroup difference was not statistically significant, the notable disparity in effect sizes suggests a potential trend wherein highly trained athletes may experience attenuated benefits. Numerous studies indicate that the efficacy of ergogenic aids is often attenuated in trained populations [7], where optimized physiological adaptations and nutritional status limit the potential for further improvement, thereby reducing the marginal benefit of supplementation [63]. Conversely, for recreationally active individuals, the comparatively larger effect size may contribute to improved exercise tolerance or reduced perceived exertion, potentially supporting higher training loads [5]. Consequently, while preliminary evidence supports the utility of CAF + CHO across recreationally active and trained levels, larger sample sizes are required to verify whether the magnitude of this ergogenic effect is modulated by training status.

### 4.4. Practical Implications

In practical terms, our findings suggest that for athletes and practitioners who routinely rely on CHO ingestion as an ergogenic aid, the addition of CAF appears to be a highly effective strategy to maximize performance outcomes. Regarding administration method, preliminary finding suggests that athletes should prioritize a CMR strategy when implementing CAF + CHO supplementation protocol, especially when gastrointestinal tolerance or time constraints limit fluid intake. Finally, these strategies are applicable to both recreationally active and trained individuals, making them broadly relevant for real-world training and competition contexts. Nevertheless, given the low certainty of evidence and the methodological heterogeneity across studies, these strategies should be implemented with careful consideration of individual characteristics, sport-specific demands, and contextual factors.

### 4.5. Future Research Perspectives

Based on the findings of this meta-analysis, several key directions for future research are warranted. First, although CAF + CHO shows promising ergogenic potential for HIIE, how specific interval types mechanistically modulate this effect remains unclear. Future studies should employ advanced assessments (e.g., blood lactate kinetics, surface electromyography, and cortical excitability) to determine whether interval-specific demands, such as rapid anaerobic turnover and muscle acidity, blunt or facilitate CAF + CHO efficacy. Second, given the superior efficacy of CAF + CMR, future research should optimize this protocol by determining the ideal solution concentration, rinse volume, and timing to maximize ergogenic benefits. Third, to advance precision sports nutrition, future trials must explicitly account for inter-individual variability by targeting determinants such as genetic polymorphisms (e.g., CYP1A_2_), sex and menstrual cycle phases, habitual CAF intake, lifestyle factors (e.g., alcohol/tobacco use), and baseline training status.

### 4.6. Strengths and Limitations

To our knowledge, this appears to be the first systematic review to apply a three-level meta-analytic framework to synthesize all performance-associated results reported in trials examining CAF + CHO supplementation during HIIE. A search was performed across four major electronic databases to identify all eligible studies. To handle dependence between multiple result values extracted derived from a single study and to reduce the risk of double counting, we used a three-level meta-analysis. The robustness of the primary and moderator findings was assessed based on sensitivity analyses. Finally, we appraised the certainty of evidence using the GRADE framework and evaluated methodological quality using the PEDro scale to support transparent interpretation and practical application.

Despite these strengths, a few shortcomings should be considered. First, only a small number of studies were included, particularly the fact that only two studies evaluated CAF + CMR, constitutes a major limitation. This scarcity of data substantially increases the risk of Type II errors, thereby constraining the stability and applicability of our conclusions. Specifically, the small number of available trials did not permit adequately powered subgroup analyses to explore whether the effects of CAF + CHO differ across exercise modalities (e.g., cycling vs. running). Second, we could not examine the influence of individual-level factors on CAF responsiveness, including genetic types, sex differences, menstrual cycle phase in female participants, and lifestyle habits (e.g., alcohol and cigarettes). These variables are known to influence caffeine metabolism and may partially explain variability in ergogenic responses [64,65,66]. In addition, the influence of habitual caffeine intake could not be examined, as not all included studies reported participants’ habitual caffeine consumption. The inconsistent reporting of this variable precluded subgroup or moderator analyses. Existing research has found that chronic caffeine consumption may lead to upregulation of adenosine receptors, potentially contributing to tolerance and attenuating the ergogenic response to acute supplementation [67]. Third, the potential for publication bias may not be entirely disregarded, especially in subgroups with few studies, where small-study effects may have distorted true effect sizes. Fourth, our search strategy was limited to articles published in English, which may have introduced a linguistic bias [68]. Finally, the ergogenic effects of CMR may be more pronounced under fasting conditions compared with the fed condition [45]. This work could not explore the influence of the fed state on the efficacy of CMR. These limitations highlight the need for caution in interpreting our findings and reinforce the value of high-quality, standardized article in this area.

## 5. Conclusions

Despite variability in the literature, findings from this study indicate that CAF + CHO significantly enhances performance in HIIE, with benefits evident in both recreationally active and trained individuals. Nevertheless, in light of the limited number of studies and female participants, these findings should be interpreted with caution. Current evidence indicates that the ergogenic effects may be primarily attributable to CAF, and CAF + CMR may represent an effective strategy to enhance HIIE performance. Overall, more rigorous, large-sample trials are needed to establish more reliable evidence-based recommendations.

## Figures and Tables

**Table 1:** Study characteristics.

Study	Sample + Age (Years) + Level + Sport Discipline	Habitual Caffeine Intake + Caffeine Withdrawal + Nutritional Status	Supplementation Protocol	HIIE Protocol	Outcomes
Roberts et al. 2010 [51]	8 M; 22 ± 3; trained rugby athletes	<100; 48; fasted	DOSE: CAF + CHO, 4 mg/kg CAF beverage + 500 mL (9% CHO beverage); PLA + PLA, 500 mL orange-flavored + aspartame; CHO, 500 mL (9% CHO beverage) TIMING: CAF, 1 h pre-exercise; PLA, 1 h pre-exercise and during rest rest intervals between blocks 1–2, 2–3, and 3–4	16 × (~20 s maximal sprinting [all-out sprints] with ~30 s active recovery till 315 s)	➀
Taylor et al. 2011 [47]	6 M; 21 ± 1; recreationally active males	non-habitual; 48; fasted	DOSE: CAF + CHO, 4 mg/kg CAF capsules + 1.2 g/kg glucose beverage; PLA + PLA, 4 mg/kg glucose capsules + flavored water; CHO + PLA, 4 mg/kg glucose capsules + 1.2 g/kg glucose beverage TIMING: CAF, 4 h and 2 h pre-exercise (4 mg/kg); CHO, 4 h, 3 h, 2 h and 1 h pre-exercise (1.2 g/kg)	Repeated 60 m cruising (80% MAS); 60 m maximal sprinting with ~30 s active recovery until exhaustion	➀
Lee et al. 2014 a [45]	12 M; 20 ± 1; recreationally active team-sport athletes	N.A.; 72; fed	DOSE: CAF + CHO, 6 mg/kg CAF capsules + 0.8 g/kg CHO beverage; PLA + PLA, PLA capsules + PLA beverage; CHO + PLA, PLA capsules + 0.8 g/kg CHO beverage TIMING: CAF, 70 min before exercise; PLA, 10 min before exercise	10 × (5 × 4 s cycling [all-out sprints] with 20 s active recovery)	➂➃➄
Lee et al. 2014 b [46]	8 F; 21 ± 1; trained team-sport athletes	50–100; 48; fed	DOSE: CAF + CHO, 6 mg/kg CAF capsules + 0.8 g/kg glucose beverage; PLA + PLA, 6 mg/kg PLA capsules + 0.8 g/kg PLA beverage; CHO + PLA, 6 mg/kg PLA capsules + 0.8 g/kg glucose beverage TIMING: CAF, 50 min pre-exercise; PLA, immediately pre-exercise	10 × (5 × 4 s cycling [all-out sprints] with 20 s recovery)	➂➃➄
Cooper et al. 2014 [48]	12 M; 23 ± 3; recreationally active team-sport athletes	N.A.; 24; fed	DOSE: CAF + CHO, 70 mL gel (100 mg CAF + 25 g CHO); PLA; 70 mL PLA gel; CHO, 70 mL gel (25 g CHO) TIMING: 1 h and immediately pre-exercise, midway of exercise protocol (70 mL gel)	4 × (11 cycles of 3 × 20 m walking [40% MAS] + 2 × 15 m sprint + 3 × 20 m running [80% MAS] + 3 × 20 m of jogging [60% MAS])	➀
Clarke. 2016 [52]	12 M; 28 ± 9; trained badminton athletes	N.A.; 12; fed	DOSE: CAF + CHO, 7 mL/kg beverage (4 mg/kg CAF + 6.4% CHO beverage); PLA, 7 mL/kg water beverage; CHO, 7 mL/kg beverage (6.4% CHO beverage) TIMING: 7 mL/kg beverage 1 h pre-exercise and 3 mL/kg beverage during the exercise	60 s intense course (all-out sprints) with 180 s active recovery till 33 min	➀
Kasper et al. 2016 [44]	8 M; 22 ± 2; recreationally active males	240 ± 162; 48; fasted	DOSE: CAF + CMR, 200 mg CAF capsules + 25 mL 10% CMR; PLA + PLA, PLA capsules + PLA drinks; CMR, 25 mL 10% CMR TIMING: CAF, 45 min and immediately pre-exercise; CMR, 25 mL 10% CMR after every sprint	Repeated 60 s running (all-out sprints) with 60 s active recovery until exhaustion	➀
Devenney et al. 2018 [42]	8 M; 23 ± 3; recreationally active males	N.A.; 24; fed	DOSE: CAF + CMR, 200 mg CAF capsule + 25 mL 6% CMR; PLA + PLA, 200 mg PLA capsules + 25 mL PLA drinks; CMR + PLA, 200 mg PLA capsules + 25 mL 6% CHO TIMING: CAF, 45 min and immediately pre-exercise; CMR, 5 s on completion every sprint	Repeated 60 s running (90% PTV) with 60 s active recovery until exhaustion	➁
Clarke et al. 2019 [49]	8 M; 21 ± 2; trained rugby athletes	N.A.; 48; fasted	DOSE: CAF + CHO, 3 mg/kg CHO beverage + 100 mL beverage (6.9% CHO); CHO, 100 mL beverage (6.9% CHO) TIMING: CAF, 3 mg/kg CAF 1 h pre-exercise; PLA, 500 mL 60 min pre-exercise + 130 mL at immediately and multiple timepoints throughout Bouts 1–2	8 × (28.5 m cruising [80% MAS]; 28.5 m maximal sprinting [100% MAS] with ~60 s active recovery till 5.36 min)	➁
Keane et al. 2020 [50]	10 M; 22 ± 2; trained Hurling athletes	N.A.; 48; fed	DOSE: CAF + CHO, 200 mg CAF capsule + 6% CHO beverage; PLA + PLA, non-caffeinated capsule + non-CHO beverage; CHO + PLA, non-CAF capsule + 6% CHO beverage TIMING: CAF, 1 h pre-exercise; CHO, consumed in three boluses during exercise: 250 mL (15 min), 350 mL (30 min), and 250 mL (45 min)	3 × (12 × 20 m running [all-out sprints] with 30 s active recovery)	➀
Fowles et al. 2021 [43]	13 M; 32 ± 11; trained cyclists	190 ± 134; 24; fed	DOSE: CAF + CHO, 16 mL/kg beverage (1.52 mg/kg CAF + 1.70 g/kg CHO); PLA, CAF-Free drink TIMING: 1 h (7 mL/kg), 50 min, 35 min and 20 min (3 mL/kg) before exercise	4 × 60 s cycling (all-out sprints) with 300 s active recovery	➂➃➄

a, b represents different studies within the same year. M, male. F, female. PTV, peak treadmill velocity. MAS, maximal aerobic speed. W_max_, maximal power. CAF, caffeine. CMR, carbohydrate mouth rinse. PLA, placebo. CHO, carbohydrate. N.A., the information was not available. The ➀, ➁, ➂, ➃, and ➄ represent exercise time, distance, mean power, peak power, and total work.

## Data Availability

The research data are available upon request from the corresponding authors.

## Supplementary Materials

The following supporting information can be downloaded at https://www.mdpi.com/article/10.3390/nu18121868/s1. Supplementary S1: Search Strategy; Supplementary S2: PEDro Assessment; Supplementary S3: GRADE Assessment; Supplementary S4: Funnel Plot; Supplementary S5: Power Visualization; Supplementary S6: Sensitivity Analysis Based on Level 2 and Level 3 (Leave-One-Out); Supplementary S7: Moderator Analysis After Excluding Outliers; Supplementary S8: Manuscript Checklist. Reference [68] is cited in the Supplementary Materials.
