# Supplementary material for: Synergy or Dominance? The Ergogenic Effects of Caffeine and Carbohydrate on High-Intensity Interval Exercise Performance: A Three-Level Meta-Analysis
Source: Nutrients. 2026 Jun 10;18(12):1868. doi: 10.3390/nu18121868 (PMC13304765; doi:10.3390/nu18121868)
Supplement: Supplementary file 1 [file nutrients-18-01868-s001.zip › nutrients-4337294-supplementary.pdf]

Title: Synergy or Dominance? The Ergogenic Effects of Caffeine and Carbohydrate on High-Intensity Interval Exercise Performance: A Three-Level Meta-Analysis

Hao Li [1,†], Yixiang Peng [2,†], Baiyu Liu [3], Li Ding [1], Kai Xu [1], Tze-huan Lei [4], Bomin Gong [5]*, Yinhang Cao [1*]

1 School of Athletic Performance, Shanghai University of Sport, Shanghai, China
2 Faculty of Health Sciences and Sports, Macao polytechnic University, Macao, China
3 School of Physical Education, Shanghai Normal University, Shanghai, China
4 College of Physical Education, Hubei Normal University, Huangshi, China
5 Department of Physical Education, Shanghai University of Traditional Chinese Medicine, Shanghai, China
† These authors contributed equally to this work.

*Correspondence:
Yinhang Cao
School of Athletic Performance
Shanghai University of Sport
Shanghai City 200438, China
Tel & Fax: +86-21-65507125
E-mail: caoyinhang@sus.edu.cn

Material Contents

Number	Material
1	Supplementary S1 (Search Strategy)
2	Supplementary S2 (PEDro Assessment)
3	Supplementary S3 (GRADE Assessment)
4	Supplementary S4 (Funnel plot)
5	Supplementary S5 (Power Visualization)
6	Supplementary S6 (A Sensitivity Analysis based on level 2 and level 3 Leave-one-out)
7	Supplementary S7 (Moderator Analysis After Excluding Outliers)
8	Supplementary S8 (Manuscript Checklist)

Supplementary S1 (Search Strategy)

Data	Query	Results
PubMed	("Caffeine"[Mesh] OR caffeine[Title/Abstract] OR caffeinated[Title/Abstract] OR coffee[Title/Abstract] OR "energy drink*"[Title/Abstract] OR "energy shot*"[Title/Abstract] OR guarana[Title/Abstract] OR methylxanthine*[Title/Abstract]) AND ("Dietary Carbohydrates"[Mesh] OR carbohydrate*[Title/Abstract] OR carb[Title/Abstract] OR carbs[Title/Abstract] OR CHO[Title/Abstract] OR glucose[Title/Abstract] OR fructose[Title/Abstract] OR sucrose[Title/Abstract] OR maltodextrin[Title/Abstract] OR sugar*[Title/Abstract] OR "mouth rins*"[Title/Abstract] OR "mouth wash*"[Title/Abstract] OR CMR[Title/Abstract]) AND ("High-Intensity Interval Training"[Mesh] OR "high intensity interval"[Title/Abstract] OR HIIE[Title/Abstract] OR HIIE[Title/Abstract] OR "sprint interval"[Title/Abstract] OR SIT[Title/Abstract] OR "repeated sprint"[Title/Abstract] OR RSA[Title/Abstract] OR "intermittent exercise"[Title/Abstract] OR "intermittent sprint"[Title/Abstract] OR "all-out"[Title/Abstract] OR supramaximal[Title/Abstract] OR Wingate[Title/Abstract] OR "team sport*"[Title/Abstract] OR soccer[Title/Abstract] OR football[Title/Abstract] OR rugby[Title/Abstract] OR basketball[Title/Abstract] OR "racket sport*"[Title/Abstract])	109

WOS	TS=(Caffeine OR caffeinated OR coffee OR "energy drink*" OR "energy shot*" OR gu arana OR methylxanthine*) AND TS=("Dietary Carbohydrate*" OR carbohydrate* OR c arb OR carbs OR CHO OR glucose OR fructose OR sucrose OR maltodextrin OR sug ar* OR "mouth rinse*" OR "mouth wash*" OR CMR) AND TS=("High-Intensity Interv al Training" OR "high intensity interval" OR HIIT OR HIIE OR "sprint interval" OR S IT OR "repeated sprint" OR RSA OR "intermittent exercise" OR "intermittent sprint" O R "all-out" OR supramaximal OR Wingate OR "team sport*" OR soccer OR football O R rugby OR basketball OR "racket sport*")	107
Scopus	TITLE-ABS-KEY (caffeine OR caffeinated OR coffee OR "energy drink*" OR "energy shot*" OR guarana OR methylxanthine*) AND TITLE-ABS-KEY ("dietary carbohydrate *" OR carbohydrate* OR carb OR carbs OR cho OR glucose OR fructose OR sucrose OR maltodextrin OR sugar* OR "mouth rins*" OR "mouth wash*" OR cmr) AND TIT LE-ABS-KEY ("high-intensity interval training" OR "high intensity interval" OR HIIT O R hiie OR "sprint interval" OR sit OR "repeated sprint" OR rsa OR "intermittent exerc ise" OR "intermittent sprint" OR "all-out" OR supramaximal OR wingate OR "team spo rt*" OR soccer OR football OR rugby OR basketball OR "racket sport*")	177

Cochrane	(("Caffeine" [MeSH Terms] OR caffeine [ti,ab] OR caffeinated [ti,ab] OR coffee [ti,ab] OR "energy drink*" [ti,ab] OR "energy shot*" [ti,ab] OR guarana [ti,ab] OR methylxanthine* [ti,ab])) AND (("Dietary Carbohydrates" [MeSH Terms] OR carbohydrate* [ti,ab] OR carb [ti,ab] OR carbs [ti,ab] OR CHO [ti,ab] OR glucose [ti,ab] OR fructose [ti,ab] OR sucrose [ti,ab] OR maltodextrin [ti,ab] OR sugar* [ti,ab] OR "mouth rins*" [ti,ab] OR "mouth wash*" [ti,ab] OR CMR [ti,ab])) AND (("High-Intensity Interval Training" [MeSH Terms] OR "high intensity interval" [ti,ab] OR HIIT [ti,ab] OR "sprint interval" [ti,ab] OR SIT [ti,ab] OR "repeated sprint" [ti,ab] OR RSA [ti,ab] OR "intermittent exercise" [ti,ab] OR "intermittent sprint" [ti,ab] OR "all-out" [ti,ab] OR supramaximal [ti,ab] OR Wingate [ti,ab] OR "team sport*" [ti,ab] OR soccer [ti,ab] OR football [ti,ab] OR rugby [ti,ab] OR basketball [ti,ab] OR "racket sport*" [ti,ab]))	131

Supplementary S2 (PEDro Assessment)

Study	Item 1	Item 2	Item 3	Item 4	Item 5	Item 6	Item 7	Item 8	Item 9	Item 10	Item 11	Total	Rating
Devenney, S et al., 2018	YES	1	1	1	1	1	0	1	1	1	1	9	Good
Fowles, J.R et al., 2021	YES	1	1	1	1	1	0	1	1	1	1	9	Good
Kasper, A.M et al., 2016	NO	1	0	1	1	1	0	1	1	1	1	8	Good
Lee, C.L et al., 2014 a	YES	1	1	1	1	1	0	1	1	0	1	8	Good
Taylor, C et al., 2011	NO	1	1	1	1	1	0	1	1	1	1	10	Excellent
Lee, C.L et al., 2014	YES	1	1	1	1	1	0	1	1	0	1	9	Good
Cooper, R et al., 2014	YES	1	1	1	1	1	0	1	1	1	1	9	Good
Clarke, J. S et al., 2019	NO	1	1	1	1	1	0	1	1	1	1	9	Good
Keane, J et al., 2020	NO	1	1	1	1	1	0	1	1	0	1	8	Good
Roberts, S. P et al., 2010	YES	1	1	1	1	1	0	1	1	1	1	11	Excellent
Clarke, N. D., 2014	NO	1	0	1	1	1	0	1	1	1	1	8	Good

Note: "excellent" (10 - 11 points); "good" 179 (7 - 9 points); "fair" (5 - 6 points); and "poor" (0 - 4 points)

1. eligibility criteria were specified (not included in the total score)
2. subjects were randomly allocated to groups (in a crossover study, subjects were randomly allocated an order in which treatments were received)
3. allocation was concealed
4. the groups were similar at baseline regarding the most important prognostic indicators
5. there was blinding of all subjects
6. there was blinding of all therapists who administered the therapy
7. there was blinding of all assessors who measured at least one key outcome
8. measures of at least one key outcome were obtained from more than 85% of the subjects initially allocated to groups

9. all subjects for whom outcome measures were available received the treatment or control condition as allocated or, where this was not the case, data for at least one key outcome was analyzed by "intention to treat"
10. the results of between-group statistical comparisons are reported for at least one key outcome
11. the study provides both point measures and measures of variability for at least one key outcome
12. did the study assess the effectiveness of the blinding to the CHO-CAF condition

Supplementary S3 (GRADE Assessment)

Outcome	K	Certainty of Evidence Assessment					Hedge's g [95% CI] *	GRADE†
		Risk of Bias	Inconsistency	Indirectness	Imprecision	Others		
**Primary Outcome**								
Exercise performance	K = 40	Serious	Not serious	Not serious	Not serious	Publication bias	0.44 [0.23, 0.66] *	⊕⊕◯◯ Low
**Administration method**								
MR	K = 4	Serious	Not serious	Not serious	Serious	None	0.91 [0.49, 1.33] *	⊕⊕◯◯ Low
Ingestion	K = 36	Not serious	Not serious	Not serious	Not serious	Publication bias	0.33 [0.14, 0.52] *	⊕⊕◯◯ Low
**Control group**								

CAF	K = 10	Serious	Not serious	Not serious	Not serious	None	0.10 [-0.09, 0.28]	⊕⊕◯◯ Low
CHO	K = 14	Serious	Serious	Not serious	Serious	Publication bias	0.32 [0.10, 0.53] *	⊕⊕◯◯ Low
PLA	K = 16	Not serious	Not serious	Not serious	Not serious	Publication bias	0.66 [0.29, 1.03] *	⊕⊕◯◯ Low
Training status								
Recreational active	K = 17	Serious	Serious	Not serious	Serious	Publication bias	0.66 [0.23, 1.10] *	⊕⊕◯◯ Low
Trained	K = 23	Not serious	Not serious	Not serious	Not serious	Publication bias	0.26 [0.09, 0.43] *	⊕⊕◯◯ Low

Supplementary S4 (Funnel plot)

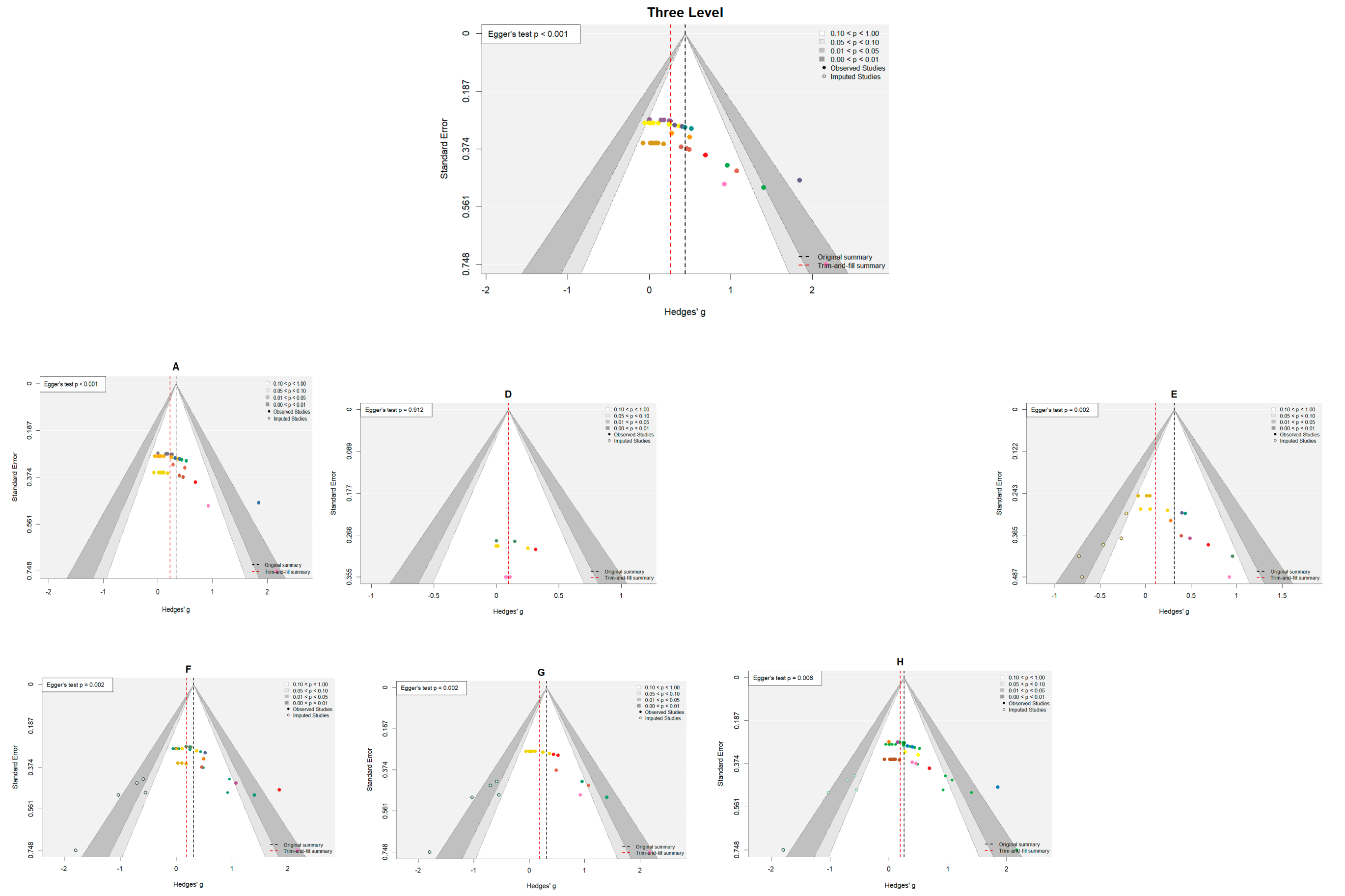

Figure S1 Funnel plot of standard mean difference against standard error. A, CHO ingestion; D, CAF control; E, CHO control; F, PLA control; G, recreationally active; H, trained.

Supplementary S5 (Power Visualization)

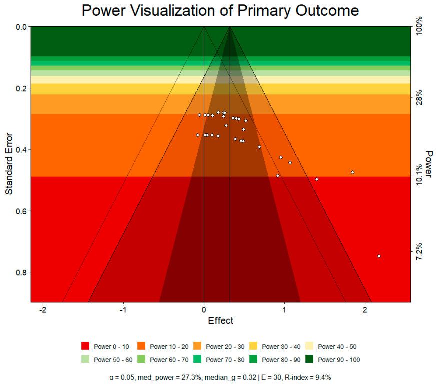

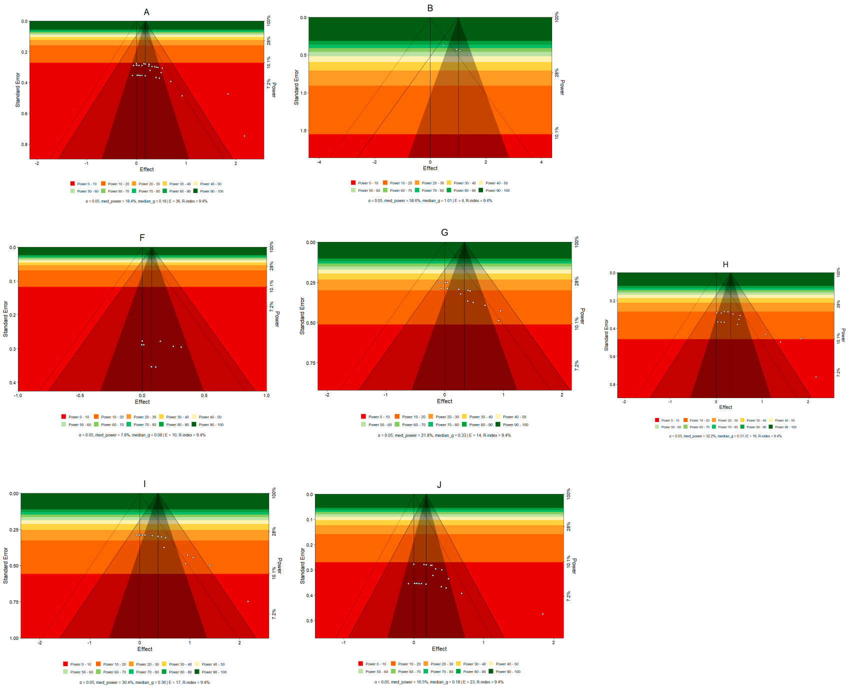

Notes: The vertical solid line represents the pooled effect size, and the vertical dash line represents the adjusted pooled effect size. Significance contours at .05 and .01 levels are noted by the shaded area. manpower indicates the median power of all included effect sizes. d33% and d66% indicate the true effect sizes necessary for achieving 33% and 66% levels of median power. E, O, and PTES show the results of a test of excess significance. R-index denotes the expected replicability of findings. A, CMR; B, CHO ingestion; F, CAF control; G, CHO control; H, PLA control; I, recreationally active; J, trained.

Supplementary S6 (A Sensitivity Analysis)

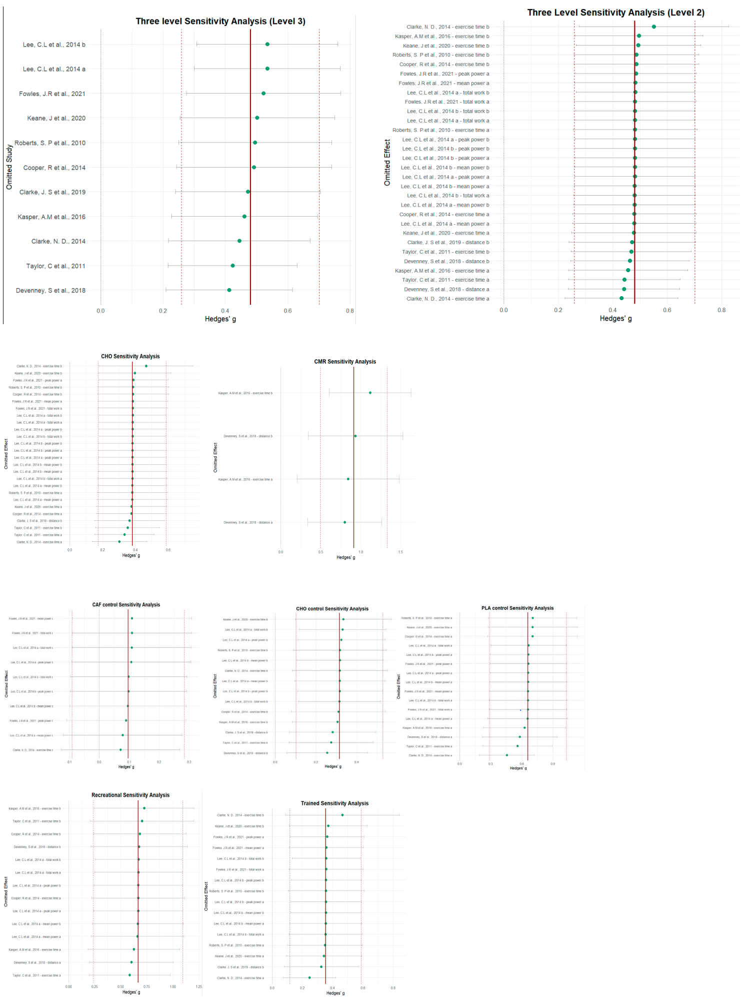

Leave-One-Out Sensitivity Analysis at the Three level analysis and Moderation analysis.

Supplementary S7 (Moderator Analysis After Excluding Outliers)

Characteristics		Forest plot	Summary of finding			
subgroup	K	Random effect model	Hedge's g (95%CI)	*p* value	I^2	$p_{\text{difference}}$
**Administration method**						0.008
CMR	2		1.01 [0.41, 1.61]	0.001	0	
CHO	33		0.18 [0.08, 0.29]	0.001	0	
**Control group**						0.345
CAF	10		0.10 [-0.09, 0.28]	0.313	0	
CHO	12		0.21 [0.02, 0.40]	0.029	8	
PLA	14		0.31 [0.14, 0.49]	0.001	0	
**Training status**						0.183
Recreationally active	16		0.58 [0.19, 0.98]	0.004	51	
trained	18		0.22 [0.07, 0.37]	0.005	0	

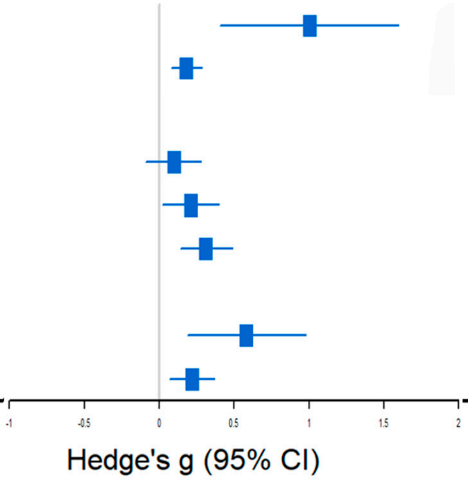

Moderator Analysis After Excluding Outliers. K, the total number of effects included in the pooled effect size; Hedge's *g*, the effect size indicators used in the pooled; 95% CI, 95% confidence interval; $p_{\text{difference}}$, statistically significant *p* values for pooled effect between moderator; *p* value, statistically significant *p* values for specific pooled effect of moderator; I^2, quantitative indicators of heterogeneity.

Supplementary S8 (Manuscript Checklist)

Section/topic	#	Checklist item	Reported on page #
**Can Blood Flow Restriction Amplify the Physiological and Performance Benefits of Interval Training in Male Intermittent-Sport Athletes? A Systematic Review and Meta-Analysis**			
Title	1	Identify the report as a systematic review, meta-analysis, or both.	Yes
**ABSTRACT**			
Structured summary	2	Provide a structured summary including, as applicable: background; objectives; data sources; study eligibility criteria, participants, and interventions; study appraisal and synthesis methods; results; conclusions and implications of key findings.	Yes
**INTRODUCTION**			
Rationale	3	Describe the rationale for the review in the context of what is already known.	Yes
Objectives	4	Provide an explicit statement of questions being addressed with reference to participants, interventions, comparisons, outcomes, and study design (PICOS).	Yes
**METHODS**			
Protocol and registration	5	Indicate if a review protocol exists, if and where it can be accessed (e.g., Web address), and, if available, provide registration information including registration number.	Yes
Eligibility criteria	6	Specify study characteristics (e.g., PICOS, length of follow-up) and report characteristics (e.g., years considered, language, publication status) used as criteria for eligibility, giving rationale.	Yes
Information sources	7	Describe all information sources (e.g., databases with dates of coverage) in the search.	Yes

Search	8	Present full electronic search strategy for at least one database, including any limits used, such that it could be repeated.	Yes
Study selection	9	State the process for selecting studies (i.e., screening, eligibility, included in systematic review, and, if applicable, included in the meta-analysis).	Yes
Data collection process	10	Describe method of data extraction from reports (e.g., piloted forms, independently, in duplicate) and any processes for obtaining and confirming data from investigators.	Yes
Data items	11	List and define all variables for which data were sought (e.g., PICOS) and any assumptions and simplifications made.	Yes
Risk of bias in individual studies	12	Describe methods used for assessing risk of bias of individual studies (including specification of whether this was done at the study), and how this information is to be used in any data synthesis.	Yes
Summary measures	13	State the principal summary measures (e.g., risk ratio, difference in means).	Yes
Synthesis of results	14	Describe the methods of handling data and combining results of studies, if done, including measures of consistency (e.g., I^2) for each meta-analysis.	Yes

**Section/topic**	**#**	**Checklist item**	**Reported on page #**
Risk of bias across studies	15	Specify any assessment of risk of bias that may affect the cumulative evidence (e.g., publication bias, selective reporting within studies).	Yes
Additional analyses	16	Describe methods of additional analyses (e.g., sensitivity or subgroup analyses, meta-regression), if done, indicating which were pre-specified.	Yes
**RESULTS**			

Study selection	17	Give numbers of studies screened, assessed for eligibility, and included in the review, with reasons for exclusions at each stage, ideally with a flow diagram.	Yes
Study characteristics	18	For each study, present characteristics for which data were extracted (e.g., study size, PICOS, follow-up period) and provide the citations.	Yes
Risk of bias within studies	19	Present data on risk of bias of each study and, if available, any outcome level assessment.	Yes
Results of individual studies	20	For all outcomes considered (benefits or harms), present, for each study: (a) simple summary data for each intervention group (b) effect estimates and confidence intervals, ideally with a forest plot.	Yes
Synthesis of results	21	Present results of each meta-analysis done, including confidence intervals and measures of consistency.	Yes
Risk of bias across studies	22	Present results of any assessment of risk of bias across studies.	Yes
Additional analysis	23	Give results of additional analyses, if done (e.g., sensitivity or subgroup analyses, meta-regression [see Item 16]).	Yes
**DISCUSSION**			
Summary of evidence	24	Summarize the main findings including the strength of evidence for each main outcome; consider their relevance to key groups (e.g., healthcare providers, users, and policy makers).	Yes
Limitations	25	Discuss limitations at study and outcome level (e.g., risk of bias), and at review-level (e.g., incomplete retrieval of identified research, reporting bias).	Yes
Conclusions	26	Provide a general interpretation of the results in the context of other evidence, and implications for future research.	Yes
**FUNDING**			
Funding	27	Describe sources of funding for the systematic review and other support (e.g., supply of data); role of funders for the systematic review.	Yes

From: [68]
